# Synergistic Inhibition of Survival, Proliferation, and Migration of U87 Cells with a Combination of LY341495 and Iressa

**DOI:** 10.1371/journal.pone.0064588

**Published:** 2013-05-27

**Authors:** Zarina Yelskaya, Vangie Carrillo, Ewa Dubisz, Hira Gulzar, Devon Morgan, Shahana S. Mahajan

**Affiliations:** Department of Health Sciences, Hunter College, City University of New York, New York, New York, United States of America; Rockefeller University, United States of America

## Abstract

Glioblastomas exploit various molecular pathways to promote glutamate- dependent growth by activating the AMPA (2-amino-3-(3-hydroxy-5-methyl-isoxazol-4-yl) propanoic acid) receptor, the group II metabotropic glutamate receptor, mGluR, and the epidermal growth factor receptor, EGFR. We hypothesized that targeting more than one of these pathways would be more effective in inhibiting glutamate-dependent growth. Using a model of U87 cell line, we show that blocking glutamate release by Riluzole inhibits cell proliferation. Glutamate-dependent growth is effectively inhibited by a combination of Iressa, an inhibitor of EGFR activation and LY341495, a group II mGluR inhibitor. Treatment of U87 cells with a combination of Iressa and LY341495 inhibits proliferation as indicated by Ki-67 staining, induces apoptosis and inhibits migration of U87 cells more effectively than the treatment by Iressa or LY341495 alone. These results demonstrate that a combinatorial therapy with Iressa and LY341495 is more effective due to synergistic effects of these drugs in inhibiting the growth of glioblastoma.

## Introduction

Glioblastoma multiforme, one of several kinds of gliomas, is a cancer of astrocytes in the brain and the spinal cord, and is the most common and malignant form of cancer of the central nervous system (CNS) [1]. Glioblastomas are high-grade (grade 4 gliomas) cancers, with a poor prognosis for patients due to their aggressive growth behavior and highly invasive nature [2], [3]. Treatment options for glioblastoma are limited to surgery, chemotherapy, and radiation with a poor survival outcome. Moreover, surgical intervention is often ineffective due to the invasive nature of the tumor [4], [5].

Many gliomas have been shown to release high levels of glutamate, which promotes malignancy [6], [7], [8]. More specifically, glutamate levels in U87 cells culture media were high due to its secretion [9]. Glutamate activates both fast-acting ionotropic and slow-acting metabotropic glutamate receptors in glial cells and is an important key regulator of invasive growth of glioblastoma [10]. Autocrine secretion of glutamate is up-regulated by cysteine-glutamate exchange [8], [11]. Additionally, low re-uptake of glutamate by loss of excitatory amino acid transporter (EAAT2) [7], [8] contributes to excess glutamate. The presence of excessive glutamate promotes invasive growth of glioblastoma cells and kills surrounding neurons due to glutamate neurotoxicity [7], [12]. Activation of ionotropic glutamate receptors, particularly that of the AMPA receptors plays a crucial role in growth and migration of glioblastoma cells [13]. AMPA receptors assemble as homo or hetero tetramers of GluR1-4 subunits and depending on subunit composition AMPA receptors form Ca^2+^-permeable (GluR1, 3, 4) or Ca^2+^-impermeable (GluR2-containing) channels. GluR2-containing AMPA receptors are Ca^2+^-impermeable due to the presence of an arginine at the pore apex, which is introduced post-transcriptionally by RNA editing [14], [15]. In the CNS, AMPA receptors in general are Ca^2+^-impermeable. However, in high-grade gliomas AMPA receptors lack GluR2 subunit forming Ca^2+^-permeable channels [16], [17]. Furthermore, glioma cells lacking GluR2 showed enhanced migration and, importantly, blocking of Ca^2+^ influx via AMPA receptor antagonist, NBQX, inhibited growth and induced apoptosis [13], [18]. Unedited GluR2 was detected in primary human glioblastoma cell lines VU-028 and VU-122 but not in U87 cell line [19]. Unedited GluR2 in neurons promotes excitotoxic death when exposed to high glutamate levels via Ca^2+^ influx and enhanced trafficking of the Ca^2+^- permeable AMPA receptors [20]. High levels of glutamate also promote cleavage of editing enzyme ADAR2 that results in lower GluR2 editing in neurons, which may lead to excitotoxic death in neurons [21]. In contrast, over expression of Ca^2+^-permeable AMPA receptors enhanced growth proliferation of glioma cells in low serum conditions [22]. Although growth of U87 cell proliferation depends both on growth factors and activation of AMPA receptor, the role of AMPA receptor in growth enhancement is evident at low serum concentrations [22]. Thus high levels of glutamate promote proliferation of glioma cells while killing neurons due to excitotoxicity [20], [21].

Ca^2+^ influx via AMPA receptors initiates Ca^2+^ signaling cascades and promotes Ca^2+^-dependent growth via the activation of protein serine threonine kinase, Akt [17]. Akt signals protein synthesis, which allows cell survival and growth, while inhibiting apoptosis, and is crucial for conversion of anaplastic astrocytoma to glioblastoma [23]. Akt activation occurs via phosphorylation of two key residues, threonine 308 in the kinase domain and serine 473 in the C terminal regulatory domain. The phosphorylation of these residues is tightly regulated. Activation of Ca^2+^permeable AMPA receptor leads to Ca^2+^-dependent activation of Akt by its phosphorylation at serine 473 by an unidentified kinase and at threonine 308 by Ca^2+^-independent mechanism in CGNH-89 glioblastoma cell line [17]. Others have shown that threonine 308 phosphorylation is activated by PDK1 [24], [25], [26], [27]. Full activation of Akt requires phosphorylation at threonine 308 and serine 473 and threonine activation is a crucial step for complete activation of Akt [28], [29].

Glutamate stimulates the up-regulation of epidermal growth factor receptor (EGFR) in glioblastoma cell line, U87 [30]. In 40–50% of glioblastomas EGFR is present at levels several-fold higher compared to the levels in normal cells [31], [32]. Epidermal growth factor receptor stimulation causes the activation of phosphatidylinositol-3-OH kinase (PI3 K), which converts membrane phosphatidylinositiol (5,5)-bisphosphate (PIP2) to phosphatidylinositol (3,4,5)-triphosphate (PIP3). PIP3 in the membrane recruits and activates Akt [33], [34]. Iressa is an EGFR inhibitor that blocks the activity of EGFR and stops cell growth and has been shown to inhibit glioblastoma cell growth [30]. Interestingly, heterodimeric EGFR receptors activate P13 K pathway and may be activating growth in glioblastoma via Akt activation [30], [35], [36], [37]. A specific inhibitor, LY294002, inhibits PI3 K and blocks phosphorylation of Akt [17]. Wortmannin inhibits phosphorylation of Akt on threonine 308 by inhibiting P13 K that activates PDK1 [29], [38].

EGFR activation in gliomas also leads to activation of the MAP kinase pathway [39]. MAP kinase pathways regulate proliferation, differentiation and survival of cells in several types of tumors including glioma [40]. ERK1 and 2 are members of the MAP kinase pathway that are regulated by phosphorylation, and have been targeted by drugs to reduce proliferation in glioma [41].

**Figure 1:**
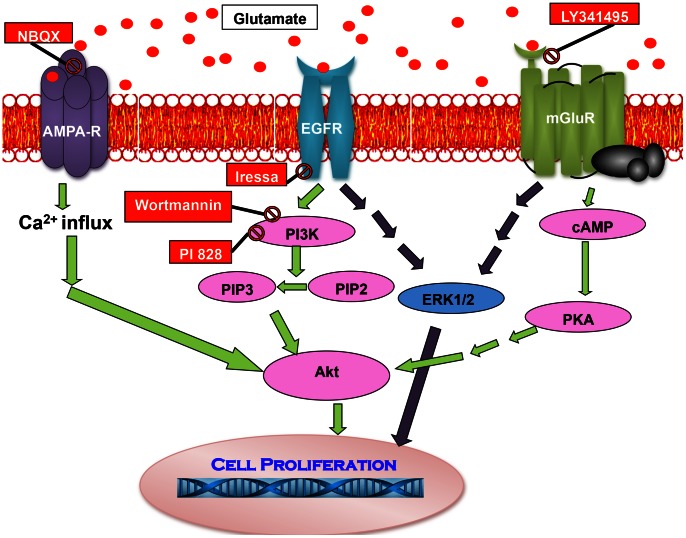
Glutamate activates several pathways to stimulate growth in glioblastoma cells. Glutamate activates Ca^2+^-permeable AMPA receptors, EGFR receptor and group II metabotropic receptors. AMPA receptor, EGFR, and mGluR are stimulated by glutamate to activate overlapping pathways for cell growth.

Glutamate stimulates growth of glioblastoma by activating the metabotropic glutamate receptors, mGluR. Drugs such as LY341495, that specifically block group II (mGluR2/3) mGluRs, are reported to inhibit the growth of glioblastoma cells *in vivo* [9], [42]. Group II mGluR in U87 cells have been shown to activate both P13 Kinase pathway and MAP kinase pathway. Furthermore, inhibition by LY341495 resulted in reduced activation of both Akt phosphorylation as well as ERK1/2 phosphorylation in U87 cells [9]. Signaling pathways stimulated by glutamate, site of action of drugs used and convergence of the signaling pathways is shown in Figure 1.

Thus, glutamate appears to stimulate multiple pathways to modulate the invasive growth of glioblastoma (Figure 1). Drugs that block the activation of these pathways, such as AMPA receptor by antagonist, NBQX, mGluR2/3 inhibitor, LY341495, EGFR inhibitor, Iressa, Akt activation inhibitors Wortmannin and LY294002 have been shown to reduce the growth of glioblastoma when used independently [9], [13], [17], [30]. Besides the activation of the glutamate receptors such as AMPA receptor, mGluR and EGFR, Akt activation is involved in the stimulation of growth by more than one pathway (AMPA receptor and EGFR receptor and mGluR activation). Furthermore, the MAP kinase pathway is activated via both EGFR and mGluR activation [9], [41], [42]. An effective treatment may necessitate the use of more than one drug for a more vehement inhibition of growth. We hypothesize, that a particular/selective combination of these drugs will work more effectively to inhibit the growth of U87, glioblastoma cells, compared to the use of individual drugs. U87 cells have been shown to secrete high levels of glutamate that aids invasive growth [11]. We report in the present study that a combination of LY341495, a selective blocker of mGluR2/3, and Iressa, an EGFR blocker, work most efficiently to inhibit both proliferation and migration of U87 cells and induced maximum apoptosis in the U87 cells. Such a drug combination may be useful in combinatorial therapy in patients with glioblastomas with EGFR overexpression.

## Materials and Methods

### Cell Culture

U87 cells were maintained in DMEM supplemented with 2 mM glutamate, 4.5% glucose, 1 mM pyruvate, 10% fetal bovine serum, 100 units/mL penicillin, and 100 µg/mL streptomycin. Cells were passaged every 4 days. Cells were maintained at 37°C with 95% air and 5% CO_2_. For experiments, cells were seeded in MEM media without glutamate, penicillin, and streptomycin, and 0.5% fetal bovine serum.

### Drug Treatments

2.5×10^4^ cells were seeded onto polylysine coated coverslips in a 24 well plate. Twenty-four hours later the cells were treated with drugs such as 5 µM NBQX, 1 µM LY341495, 2 µM PI828. PI282 is a more potent form of LY294002 [43]. 5 µM Wortmannin, 25 µM Iressa alone was used or in combination as specified in each experiment for 72 hours. All drugs were purchased from R&D systems, Minneapolis, MN.

### Proliferation Assay

Post-drug treatment, the cells were fixed in 4% paraformaldehyde for 10 min and permeabilized in 0.2% Triton X-100 for 5 min. The cells were washed 3 times with 1X Phosphate buffered saline (PBS) and then blocked with 10% bovine serum albumin made in 1X PBS. Immunocytochemistry was carried out using primary antibodies such as anti-mouse β III tubulin and anti-rabbit Ki-67 followed by Alexa 488-conjugated anti-rabbit secondary antibody and Alexa 568-conjugated anti-mouse antibody. The samples were mounted in mounting media (Molecular Probes) containing DAPI for nuclear staining. Images were captured at 20X magnification using a Zeiss fluorescence microscope. The images were used for counting DAPI positive and Ki-67 positive cells. At least five images were used from each sample and the experiments were repeated at least four times. The average obtained from all experiments is presented as total cell numbers.

### TUNEL Assay

TUNEL assay was performed using the *In situ* cell death detection kit from Roche as per manufacturer’s instructions. Briefly, after cells were fixed with 4% paraformaldehyde and permeabilized in 0.2% Triton X-100, the TUNEL assay was carried by incubating the fixed cells with the TUNEL reagent containing TMR red labeled nucleotides at 37°C for 1 hour. The samples were washed in 1X PBS and mounted in mounting media containing DAPI. Fluorescent images were captured using a Zeiss fluorescence microscope at 20X magnification. The total number of DAPI positive cells and total number of TUNEL positive cells were counted from at least five images from each sample. Each experiment was repeated four times.

### Scratch Assay

Scratch assay was carried out as described by Goldberg and Kloog [44]. Briefly, 1 million cells/well were seeded on poly-lysine coated 6-well dishes. After 24 hours media was replaced with glutamate free low serum media (0.5% FBS) containing drugs. After 24 hours, three scratches were made and the media was replaced with fresh media containing drugs. Images were captured at 10X with a phase contrast inverted microscope at 0, 6, 18 and 24 hours. Each sample had three scratches and the experiment was repeated three times. Gap width was calculated by taking 50 measurements from each scratch to obtain the average width. Each measurement was taken from the left edge of the scratch to the right edge of the scratch going from top of the image to the bottom of the image using Image J. Total 150 measurements were obtained from one sample in each experiment to obtain the average width of the sample. Distance travelled by the cells into the gap was calculated as arbitrary units using the average width distance difference in samples at 0, 6, 18 and 24 hours.

### Data Analysis

Statistical analysis was carried out using Microsoft Excel 2010. One-way ANOVA was performed to test differences between control and different drug-treated groups. Significance was established at p-values <0.05. To test differences within groups, Tukey's post-hoc analysis was carried out using Prism software, GraphPad Software, Inc., La Jolla, CA. Significance was established at p-values <0.05.

## Results

### Dosage Curve of Glutamate on U87 Cell Growth

**Figure 2:**
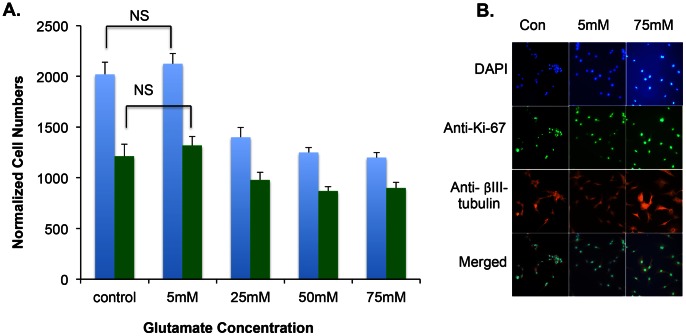
Glutamate Dosage Curve on U87 cells. Serum starved U87 cells were treated with 5 mM to 75 mM concentrations of glutamate in the media for 72 hours and the cells were fixed and stained with anti-rabbit Ki-67 antibody and anti-mouse β III tubulin antibody along with DAPI stain for nuclei. ALEXA 488-conjugated anti-rabbit antibody and Alexa 568-conjugated anti-mouse antibodies were used as secondary antibodies. Images were captured at 40X using a Zeiss fluorescence microscope. **A.** Total number of cells counted and average of 4 independent experiments based on DAPI staining, blue bars and Ki-67 positive cells are shown as green bars. No significant (Not Significant, NS) differences were found between the control and the sample treated with 5 mM glutamate for both DAPI and Ki-65 staining. **B.** Representative images of DMSO treated control or samples treated with 5 mM and 75 mM glutamate.

We aimed to study inhibition of glutamate-stimulated growth of U87 cells. To demonstrate the effect of dose concentration of exogenous glutamate on U87 cells in culture we used a proliferation assay as described in Materials and Methods. At 5 mM glutamate concentration, the growth represented by DAPI staining (blue bars) was similar to untreated control and cells showed similar morphological features when compared with the control sample. In contrast, at higher concentrations (25 mM, 50 mM or 75 mM glutamate), there was a significant reduction in the total number of cells stained by DAPI (blue bars, Figure 2 A). The cells that survived high glutamate exposure at 50 mM and 75 mM of glutamate showed large and flattened morphology by β III tubulin staining (shown in red in Figure 2B). U87 cells show heterogeneity in culture, where some cells grow in spheres with rounder morphology while some grow as large flattened cells. Interestingly, cells at both low and high glutamate concentration showed Ki-67 staining (represented by green bars, Figure 2A). Ki-67 staining labels the proliferating cells and high glutamate concentrations seem to select a certain population of cells as shown in Figure 2B. Notably, with 5 mM, the cells are smaller and rounder whereas, with 75 mM, the cells are flatter and larger. These morphological differences between samples treated with 5 mM and 75 mM are evident by β III tubulin staining in Figure 2B. Interestingly, cells selected by high glutamate concentration are also positive for proliferation marker, Ki-67. There were no significant differences in the DAPI or Ki-65 staining between control and samples treated with 5 mM glutamate. Based on these data, we infer that U87 cells maybe secreting enough glutamate for their growth in the media, and hence are not responding to exogenously added glutamate.

### Riluzole Blocks Glutamate-dependent Growth of U87 Cells

**Figure 3:**
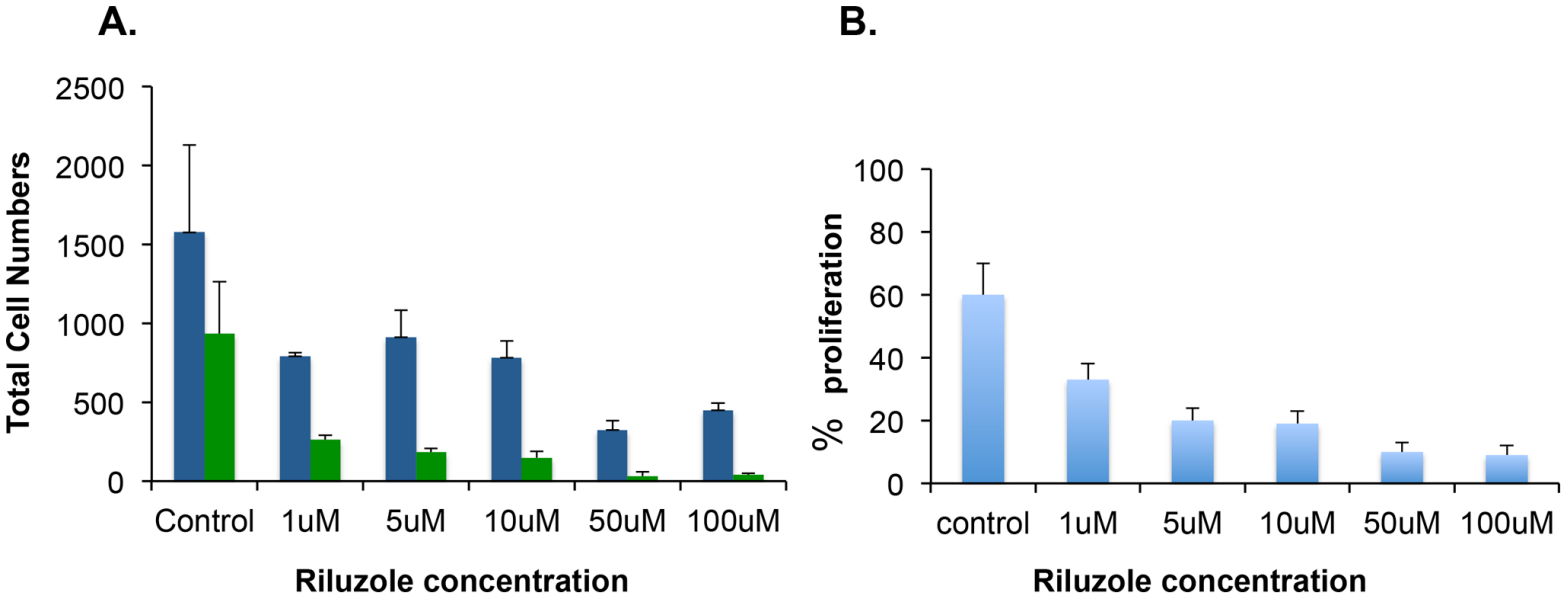
Riluzole inhibits cell proliferation in U87 cells. **A.** Proliferation was measured in the presence of Riluzole at various concentrations as indicated. The blue bars represent total number of DAPI positive cells and the green bars represent total number of Ki-67 positive cells. **B.** The percentage of Ki-67 positive cells of the total DAPI positive cells in untreated control or at various concentrations of Riluzole. The inhibition by Riluzole occurs at all doses and is significantly different when compared to the control sample, p value 0.05 by ANOVA.

Exogenous glutamate addition to U87 cultures did not result in increased cell proliferation, therefore, we wanted to test if U87 cells were secreting sufficient glutamate to stimulate their growth. We used Riluzole, a drug that blocks the secretion of glutamate and enhances the uptake of glutamate from the extracellular space thereby, reducing the effective concentration of glutamate in the media [45]. To assess proliferation, DAPI stained cells were used to score total cells and proliferating cells by counting Ki-67 positive cells. The results in Figure 3A show DAPI positive cells as blue bars and Ki-67 positive cells as green bars. Figure 3B shows percentage of proliferating cells based on total number of Ki-67 positive cells out of total number of DAPI positive cells. Compared to the control sample Riluzole treated samples showed decreased number of both DAPI positive and Ki-67 positive cells as the concentration of Riluzole increased from 1 µM to 100 µM. Figure 3B shows that in control sample 60% of the DAPI positive cells were positive for Ki-67 marker. In contrast, at 1 µM Riluzole only 30% of the DAPI positive cells were Ki-67 positive. The percentage of Ki-67 positive cells decreased in a dose-dependent manner up to a dose of 50 µM and remained same at 100 µM Riluzole (Figure 3B). Thus, the percentage of proliferating cells declined with increase in Riluzole concentration and Riluzole treated samples were significantly different when compared to untreated control sample with a p value of less than 0.05. Our results demonstrate that Riluzole inhibits proliferation of U87 cells in a dose-dependent manner, suggesting that the absence of glutamate from media prevents glutamate-dependent proliferation. These results suggest that there is enough glutamate secreted in the media for growth of U87 cells and Riluzole blocks the release of glutamate in the media.

### Iressa in Combination with LY341495 or PI828 is Effective in Inhibiting Proliferation

**Figure 4:**
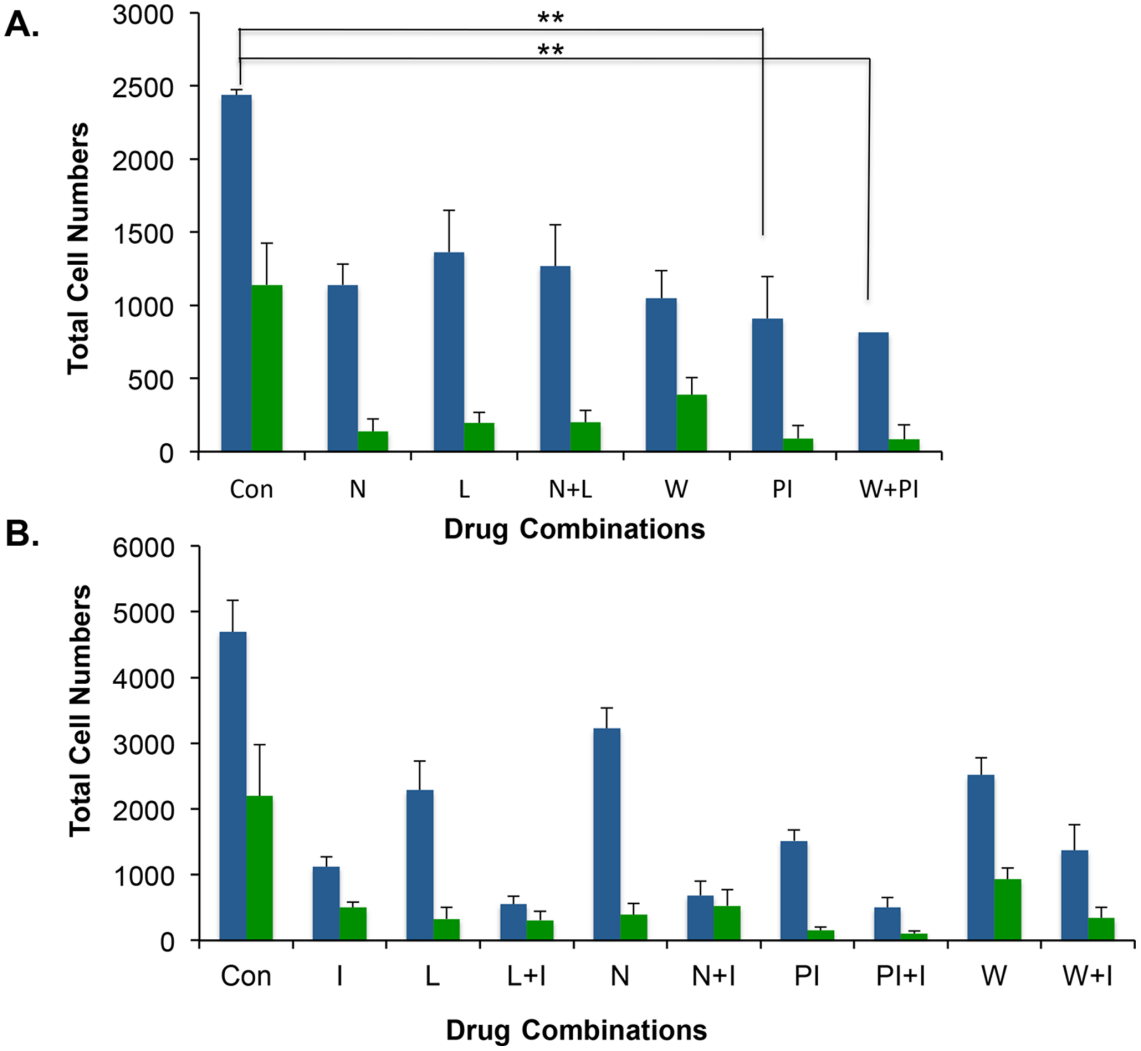
Iressa plus LY341495 inhibit proliferation of U87 cells. **A.** Proliferation assay was performed either in vehicle treated or samples treated with NBQX, 5 µM, LY341495 1 mM, NBQX+LY341495, Wortmannin 5 µM, PI828 2 µM or Wortamannin+PI828. Total number of DAPI positive cells is represented as blue bars and Ki-67 positive cells are represented as green bars. **B**. Proliferation assay was carried using Iressa 25 µM or combination of Iressa with NBQX or LY341495 or PI828 or Wortmannin as shown. Blue bars represent DAPI positive cells and green bars represent Ki-67 positive cells. DAPI staining in both 3A and 3B, control vs. PI was significantly different (p value <0.05, **) while others (N, L, W vs. control were not significant). DAPI staining in 3B control vs. N+I, control vs. L+I, control vs. W+I and control vs. P+I were significantly different (p value <0.05). For Ki-65 staining in Figure 3A, differences in control vs. drug treated samples were significant with a p value of <0.05. In Figure 3B all drug treated samples against control were significantly different. More importantly L+I or PI+I are significantly different from N+I or W+I with a p value of <0.05 with Tukey’s Multiple Comparison Test.

It is well established that U87 cells proliferate in a glutamate-dependent manner and drugs such as NBQX, an AMPA receptor blocker or LY341495, an inhibitor of metabotropic glutamate receptor have been shown to inhibit proliferation of glioma cells [9], [13]. We investigated the efficacy of combinations of different classes of drugs in inhibiting the proliferation of U87 cells. The samples were treated with DMSO, NBQX, LY341495, NBQX+LY341495, Wortmannin, PI828 or Wortmannin plus PI828. The total number of cells was scored by DAPI staining and proliferating cells were scored by Ki-67 staining. Compared to the control sample, all treatments showed decreases in total number of cells but the differences were not significant. Significant differences were observed between control and all drug treated samples for Ki-67. As shown in Figure 4A, a combination of NBQX and LY341495 was no more effective than NBQX or LY341495 alone in inhibiting proliferation as indicated by green bars. A combination of two kinase inhibitors, PI828 and Wortmannin, was more efficaciously than PI828 or Wortmannin alone in reducing the total number of cells (blue bars). However, no change was observed in the percentage of proliferating cells when compared to PI828 alone (indicated as green bars, Figure 4A). We then tested NBQX, LY341495, PI828, or Wortmannin in combination with Iressa, an inhibitor of epidermal growth factor receptor (EGFR). Treatment of cells with Iressa alone resulted in decreased proliferation compared to the untreated control as shown in Figure 4B. Furthermore, Iressa in combination with NBQX, LY341495, PI828, or Wortmannin decreased the total number of cells when compared to the individual drugs alone as shown as blue bars in Figure 4B. Significantly, Iressa, decreased the total number of cells in combination with LY341495, NBQX, or with PI828. Moreover, Iressa also decreased the number of proliferating cells when used in combination with PI828 or LY341495 shown as green bars in Figure 4B. The results in these experiments demonstrate that combination of Iressa with LY341495 or PI828 is more effective in inhibiting the proliferation of cells compared to the individual drugs alone. The differences in the proliferation of cells in samples treated with individual drugs (Iressa, LY341495, PI828) versus Iressa plus LY341495 or Iressa plus PI828 was significant with a p value of less than 0.05.

### Iressa in Combination with LY341495 Induces Maximal Apoptosis in U87 Cells

**Figure 5:**
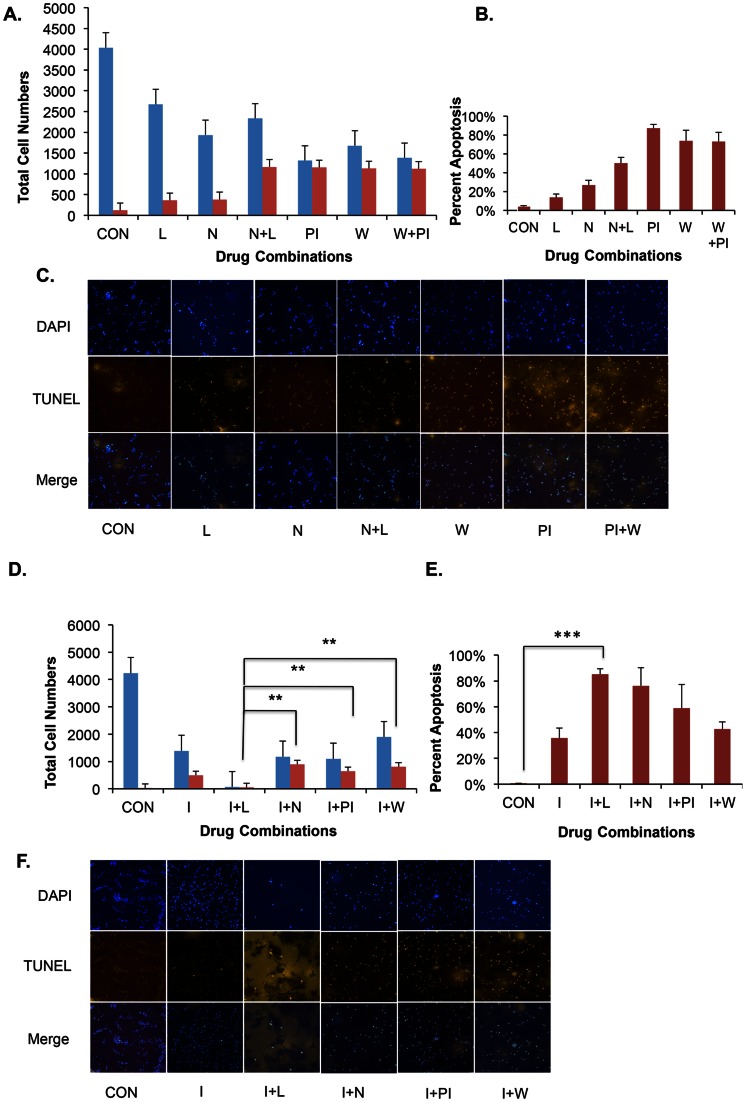
Iressa plus LY341495 induces apoptosis in U87 cells. Apoptosis was measured using TUNEL assay in samples either treated with vehicle for control or treated with LY341495, NBQX, NBQX+LY341493, PI828, Wortmannin or PI828+Wortmannin. **A.** Blue bars represent DAPI positive cells and red bars represent TUNEL positive cells. **B.** Percentage of TUNEL positive cells in each sample. **C.** Representative images of the TUNEL assay. Apoptosis was measured using TUNEL assay in samples either treated with vehicle for control or treated with Iressa or Iressa with LY341495 or NBQX or PI828 or Wortmannin. **D.** Blue bars represent DAPI positive cells and red bars represent TUNEL positive cells. **E.** Percentage of TUNEL positive cells in each sample. **F.** Representative images of the TUNEL assay. For 4A DAPI, no significant differences were found between samples except in control vs. PI and control vs. W+PI (p value <0.05). For TUNEL assay, no significant differences were found between any two samples using Tukey’s Multiple Comparison Test. In Figure 4D with DAPI staining, significant differences exist between control vs. I, control vs. I+L, control vs. I+N, control vs. I+PI, and control vs. I+W with a p value of <0.05. Significant differences were seen between I vs. I+L, I+L vs. I+N, I+L vs. I+PI and I+L vs. I+W (p value <0.05). For TUNEL positive cells significant differences were found between control vs. I, control vs. I+N, control vs. I+PI and control vs. I+W. No difference was obtained when control was compared to I+L. Significant differences were obtained between I+L vs. I+N, I+L vs. I+PI, and I+L vs. I+W (p value <0.05). Figure 4E. The percentage apoptosis is highly significant between the control and I+L samples with a p value <0.0005 (***).

Some drugs such as NBQX have been shown to inhibit growth by inducing apoptosis in CGNH-89 cells [13]. We tested the effect of combination of drugs in inducing apoptosis in U87 cells using a TUNEL assay. Total number of cells were scored by DAPI staining and apoptotic cells were scored by TUNEL staining. Representative images from the experiments are shown in Figure 5C and 5F. Percentage of apoptotic cells was calculated using total DAPI positive cells and total TUNEL positive cells from four independent experiments. Treatment with NBQX alone or LY341495 alone induced increased apoptosis compared to untreated control Figure 5A. A combination of NBQX and LY341495 induced an increased percentage of apoptosis when compared with either NBQX or LY341495 alone (Figure 5B). Treatment with PI828 or Wortmannin resulted in increased apoptosis compared to untreated control, however, the samples treated with both PI828 and Wortmannin together did not further increase the percent of apoptosis compared to that of PI828 or Wortmannin alone as shown in Figure 5A and 5B. Samples were also treated either with Iressa alone or Iressa in combination with LY341495, NBQX, PI828 or Wortmannin (Figure 5D–F). Treatment with Iressa resulted in increased apoptosis compared to control. Interestingly, treatment of samples with Iressa in combination with PI828 or Wortmannin did not increase apoptosis in cells but was less effective when compared to treatment with PI828 or Wortmannin as shown in Figure 5E and 5B. However, treatment of samples with Iressa and NBQX or Iressa and LY341495 increased apoptosis compared to Iressa alone, NBQX alone or LY341495 alone (Figure 5E and 5B). Maximum percentage of apoptosis was observed in the Iressa and LY341495 treated sample. Significant differences were found between Iressa plus LY341495 and Iressa plus NBQX or Iressa plus PI828 or Iressa plus Wortmannin. Curiously, the differences between the control sample and sample treated with Iressa plus LY341495 were not significant for TUNEL positive cells. This is due to a huge loss of cells by apoptosis in Iressa plus LY341495 treated sample. On the contrary, percentage of apoptotic cells between the control and Iressa plus LY341495 treated samples were highly significant. Control samples showed fewer number of TUNEL positive cells just like in Iressa plus LY341495. However, the number of DAPI positive cells in control far exceeds the number of DAPI positive cells in Iressa plus LY341495. These results suggest that the most effective drug combination treatment to achieve maximum apoptosis is with Iressa plus LY341495.

### Iressa in Combination with LY341495 Blocks Migration of U87 Cells

**Figure 6:**
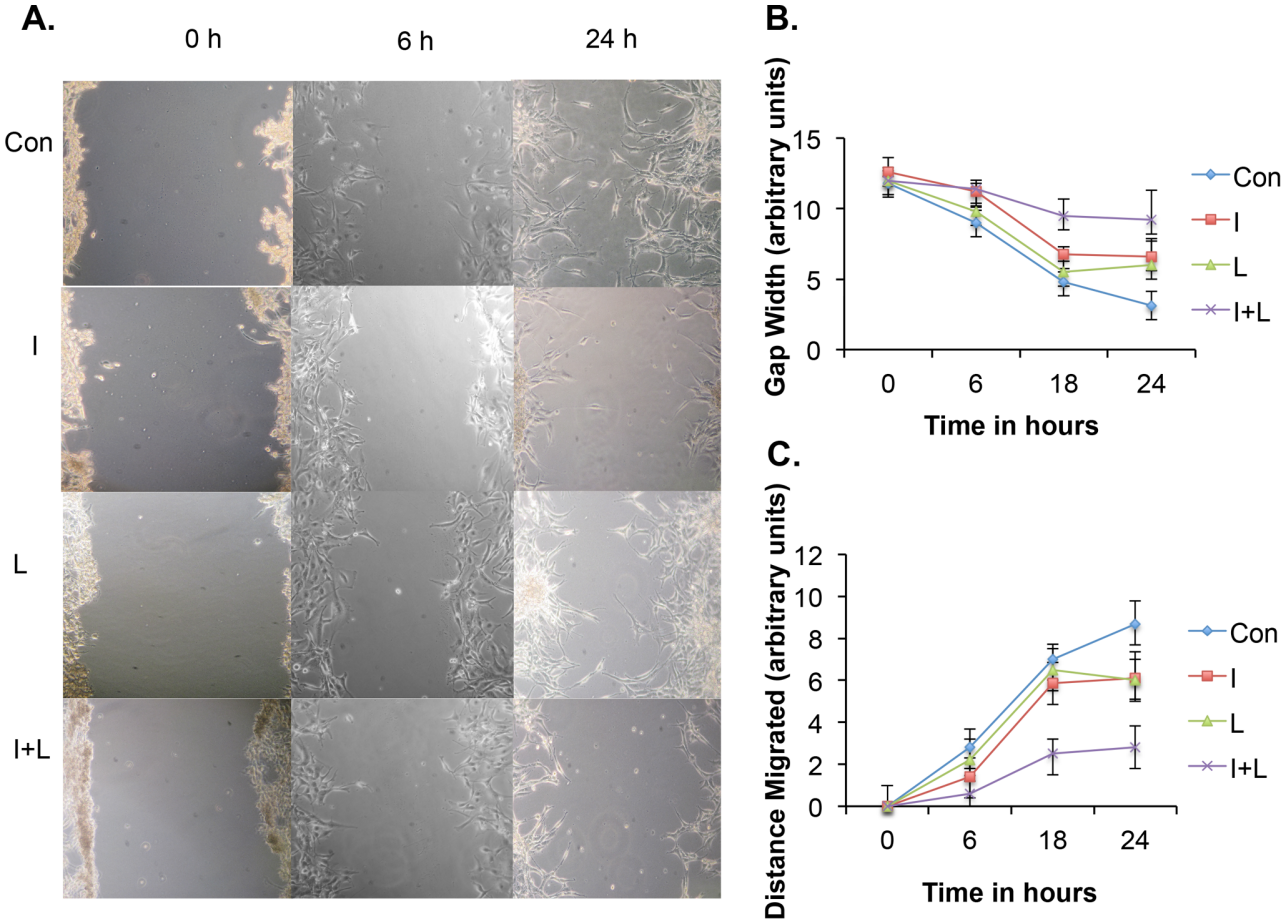
Iressa plus LY341495 inhibit U87 cell migration. Scratch assay was used to measure migration of U87 cells. Cells were either treated with vehicle for control or treated with 25 µM Iressa, 1 µM LY341495, or Iressa+LY341494, then scratched and images of the gap created were captured at 0 h, 6 h, 18 h and 24 h using phase contrast inverted scope. **A.** Representative images of the scratch in samples at 0 h, 6 h and 24 h. **B.** The gap distance was measured from 3 scratches in each sample at 0 h, 6 h, 18 h and 24 h using NIH image J. **C.** The distance travelled by the cells was calculated based on decrease in gap width distance at 0 h, 6 h 18 h and 24 h. Samples treated with Iressa plus LY341495 showed significantly more inhibition of cell migration when compared to samples treated with Iressa alone or LY341495 alone p value <0.05.

Glioblastoma cells have been shown to migrate in response to glutamate release and blocking XC-mediated glutamate release disrupted glioma invasion [11]. Migration of U87 cells under untreated conditions and drug treated conditions were tested using a scratch assay. We performed the scratch assay as described in the Materials and Methods section. Figure 6A shows representative images from each sample that was used to collect and analyze data. Figure 6B shows the average gap width in each sample at 0, 6, 18 and 24 hours. The control sample showed an average width of 5 and less than 4 arbitrary units at 18 and 24 hours respectively. In samples treated with Iressa alone, the average gap width was 7 at 18 and 24 hours. Treatment with LY341495 resulted in the gap width of 7 and 7.5 at 18 and 24-hour time points. Samples treated with both Iressa and LY341495 showed an average width of around 10 units. The distance migrated by cells was significantly reduced in samples treated with Iressa and LY341495 compared to samples treated with Iressa alone or LY341495 alone or control sample (Figure 6C). The inhibition of cell migration in Iressa plus LY341495 treated sample may be due to both inhibition of proliferation and onset of apoptosis. There were more rounded dead cells in the Iressa plus LY341495 samples when compared to control or Iressa alone, or LY341495 alone (data not shown). These results demonstrated that a combination of Iressa and LY341495 works synergistically in blocking the migration of U87 cells.

## Discussion

We examined the effectiveness of drugs targeting various pathways involved in glutamate-dependent growth, such as, AMPA receptor-mediated, mGluR-activated, EGFR-dependent pathways. We tested the effectiveness of combination of drugs targeting above pathways. Drugs targeting EGFR and mGluR have been shown to block proliferation of U87 cells [9], [30]. Our results suggest that a combination of LY341495, mGluR blocker, and Iressa, EGFR blocker is effective in preventing proliferation of U87 cells and in inducing apoptosis in about 90% of the cells. LY341495 plus Iressa is also very effective in inhibiting migration of U87 cells. The synergistic effect of Iressa and LY341495 may be due to the inhibition of converging and overlapping pathways such as Akt-1 and MAP kinase pathways. These two pathways have been shown to be utilized both by EGFR and mGluR upon activation by glutamate [9], [39], [41], [42].

In the present study, we show that higher glutamate exposure to U87 cells results in selection of flattened and enlarged cells. These enlarged and flattened cells were positive for the proliferation marker Ki-67. This result may suggest that high glutamate selection may be based on the differences in glutamate tolerance by these cells. It is possible that the glutamate receptors including NMDA and AMPA receptor expression in these cells may be different compared to those of glutamate susceptible cells and needs to be further investigated. We show that Riluzole treatment results in the inhibition of proliferation of U87 cells. Riluzole has been shown to block proliferation, invasion and migration of melanoma cells [46]. It is interesting to note that Riluzole in prostrate cancer lines has been shown to block DNA synthesis and induce apoptosis via the induction of ER stress [46]. Furthermore, in breast cancer Riluzole has shown promising results by inhibiting proliferation [47]. However, the mechanism by which the lack of, or low, glutamate inhibits proliferation in U87 cells may be due to lack of stimulation of AMPA receptor, mGluR or EGFR pathways. Further investigation is needed to study the mechanism by which Riluzole is inhibiting proliferation of U87 cells.

Ca^2+^-permeable AMPA receptors are expressed in U87 cells, which lack subunit GluR2 [13]. GluR2 subunit, which is normally edited in healthy brain when incorporated into AMPA receptor forms Ca^2+^-impermeable channels. Overexpression of GluR2 in U87 cells has been shown to inhibit proliferation by inactivation ERK1/2-Src pathway [48]. GluR2 is unedited in some glioblastomas, which results in Ca^2+^-permeable AMPA receptors [49]. AMPA receptor channels either containing unedited GluR2 or lacking GluR2 form Ca^2+^-permeable channels and such channels are involved in invasive and aggressive growth behavior in glioblastoma [13], [49]. The AMPA receptor blocker NBQX is well documented to block proliferation and migration of glioblastoma cells [13], [18]. However, we did not detect significant inhibition by NBQX even at higher concentrations (50 µM or 100 µM data not shown) or in combination with LY341495. Higher concentration of the drug resulted in precipitation of the drug in the cultures. It has been reported earlier in animals to cause nephrotoxicity due to precipitation of the drug in the kidneys [50]. The lack of adequate inhibition effect by NBQX may be due to a lower concentration of the drug (5 µM) or maybe due to partial or complete non-functionality of AMPA receptors in U87 cells where the subunits such as GluR1, GluR2 and GluR3 although expressed may not assemble into functional AMPA receptor channels [19]. Ca^2+^ influx through the AMPA receptors activates PI3K independent phosphorylation of Akt at Serine 473, which promotes proliferation and migration of CGNH89 cells [17]. Data suggests that inhibition by NBQX is not the most effective way to block cell proliferation of U87 cells. Rather, a combination of LY341495 and Iressa that target both mGluR and the EGFR is most effective in blocking proliferation of U87 cells.

PI828 and Wortmannin, both inhibitors of PI3K, showed differences in their ability to block proliferation of U87 cells. PI828 was more effective than Wortmannin in blocking proliferation. This difference may be explained based on studies that have demonstrated reduced specificity of LY294002 as a PI3K inhibitor compared to Wortmannin [51], [52], [53]. Our data shows that PI828 is more effective in combination with Iressa when compared to Iressa plus Wortmannin in inhibiting proliferation.

Combination of LY341495 plus Iressa resulted in decreased proliferation and cell survival based on the DAPI numbers (Figure 4B). Combination of LY341495 plus Iressa also induced maximal apoptosis in almost 90% of the cells when compared to a 40% by Iressa and 10% by LY341493 individually (see Figure 5E and 5B). We believe that this combination acts synergistically, blocking proliferation and inducing apoptosis, and may also be effective *in vivo* due to high penetration of LY341495 into brain and adequate levels of Iressa in the brain [9], [54].

A large number of glioblastomas show EGFR amplification [31], [32] and the combination of Iressa and LY341495 may be suited to block the proliferation of many tumors with EGFR misregulation. AMPA receptor activation and EGFR activation target the same signaling molecules, such as Akt. However, the kinase involved in the activation of Akt, at least at the S473 site may be different depending on path of activation [17]. Our data suggests that a more positive feedback loop exist between mGluR and EGFR activation in U87 cells, because a combination of inhibition of mGluR and EGFR is more effective than combination of inhibition AMPA receptor and EGFR inhibition. The synergism exhibited by Iressa and LY341495 may stem from the fact that they inhibit activation of Akt-1 and ERK1/2 activation via the inhibition of EGFR and mGluR activation [9], [39], [41]. Depending on the pathways activated in different gliomas, the combination of inhibitors that block growth proliferation and induce apoptosis may be different. Conclusively, we demonstrated that a combinatorial drug treatment with Iressa and LY341495 acts synergistically in inhibiting glutamate-dependent proliferation, inducing apoptosis and inhibiting migration of U87 cells. We believe that the synergism is due to convergence of two pathways namely the Akt pathway and the MAP kinase pathway and both pathways are activated by EGFR and mGluR upon glutamate-dependent activation.
